# Study of Changes in Optic Nerve Sheath Diameter Following Tracheal Intubation Using Macintosh Laryngoscope or Fibreoptic-Guided Intubation Through Ambu Aura-I: A Randomised Controlled Study

**DOI:** 10.7759/cureus.19782

**Published:** 2021-11-21

**Authors:** Daljinder Singh, Akashdeep Singh, Ashim Sharma, Kuldip Sandhu

**Affiliations:** 1 Department of Orthopedics, Government Medical College, Patiala, IND; 2 Department of Anesthesia, Government Medical College, Patiala, IND

**Keywords:** macintosh laryngoscope, fiberoptic intubation, fiberoptic bronchoscopy, endotracheal intubation, optic nerve sheath

## Abstract

Background: Neuroanaesthesiologists are faced with managing and optimising the intracranial pressure in the perioperative period. Laryngoscopy and tracheal intubation are known to increase sympathetic activity that is well tolerated by healthy patients but may be detrimental to many comorbid patients. We, therefore, hypothesised that airway management and tracheal intubation through Ambu Aura-I (Ambu, Baltorpbakken 13, Denmark) may be associated with lesser changes in optic nerve sheath diameter (ONSD) compared to conventional tracheal intubation and designed a study to ultrasonographically measure the changes in optic nerve sheath diameter following tracheal intubation using Macintosh laryngoscope or fibreoptic-guided intubation through Ambu Aura-I in patients receiving endotracheal anaesthesia.

Material and methods: This randomised controlled hospital-based clinical study was conducted on 60 patients divided into two groups: group 1 (n=30, tracheal intubation facilitated by direct laryngoscopy with Macintosh laryngoscope) or group 2 (n=30, fibreoptic-guided tracheal intubation through Ambu Aura-I), undergoing elective surgery under general anaesthesia requiring tracheal intubation.

Results: Baseline parameters before induction of anaesthesia were recorded for further comparison. Baseline ONSD at 3 mm behind the globe in both eyes (before induction of anaesthesia), both in transverse and the coronal plane, was measured by transorbital sonography with the patient lying in the supine position using a portable Sonosite Turbo-M ultrasonography (Fujifilm Sonosite, Bothell, USA) machine. End-tidal carbon dioxide concentration (EtCO_2_) was also recorded at this time. Observations of HR, systolic blood pressure (SBP), diastolic blood pressure (DBP), mean blood pressure (MBP), oxygen saturation (SpO_2_), EtCO_2_, and ONSD measurements were recorded immediately and at three and five minutes after intubation, and complications were recorded. Data collected were tabulated, and statistical analysis was done using SPSS 22.00 for windows (SPSS Inc, Chicago, USA). The ONSD increase peaked at 4.19±0.35 and 4.16±0.31 mm in right and left eyes. Like in group 1, the ONSD decreased slightly to 4.06±0,29 and 4.05±0.29 mm in right and left eyes in group 2 at 10 minutes after intubation. The changes in ONSD when compared to baseline values (before intubation) were statistically not significant (p>0.05). Between-group comparison in ONSD in both the eyes at different time intervals was statistically not significant (p>0.05).

Conclusion: We conclude that fibreoptic-guided tracheal intubation through Ambu Aura-I is not superior to tracheal intubation using direct laryngoscopy with Macintosh laryngoscope in terms of its effect on intracranial pressure, as measured ultrasonographically by optic nerve sheath diameter.

## Introduction

Neuroanaesthesiologists are faced with managing and optimising the intracranial pressure in the perioperative period. Airway management using tracheal intubation offers a stumbling block in the initial portion of this journey. Laryngoscopy and tracheal intubation are known to increase sympathetic activity that is well tolerated by healthy patients but may be detrimental to patients with preexisting ischemic or hypertensive heart disease. Laryngoscopy and tracheal intubation are also known to be associated with an increase in intraocular pressure (IOP) and intracranial pressure (ICP). The acute increase in IOP may be dangerous for patients with impending perforation of the eye, perforating eye injuries and glaucoma. An increase in ICP is deleterious in those having a history of neurotrauma, preexisting raised intracranial pressure, and space-occupying lesions of the brain [1]. This is due to the sympathetic cardiovascular responses to laryngoscopy which causes substantial distortion of soft tissues to bring the laryngeal inlet in the line of sight. Further, tracheal intubation is also associated with pressor responses (increase in heart rate and blood pressure). Such pressure responses are, however, minimal when laryngeal mask airway (LMA) is used for airway management instead of tracheal intubation since it does not involve the use of a laryngoscope [2]. The LMA, however, is not suitable for neurosurgical anaesthesia for brain tumour removal because of several limitations. The second-generation newer LMAs are suitable as independent ventilator devices and are also suitable as a conduit for blind or fibreoptic-guided intubation through it [2,3]. Airway management and tracheal intubation using such devices may result in minimal pressure responses since laryngoscopy is completely obviated. The Ambu Aura-I (Ambu, Baltorpbakken 13, Denmark) is a recently introduced supraglottic airway device available in different sizes with integral features designed to facilitate fibreoptic-guided tracheal intubation [3]. It includes a preformed anatomical curvature with a wider lumen which is designed to allow passage of an appropriately sized tracheal tube. There is a paucity of literature on successful fibreoptic-guided tracheal intubation through the Ambu Aura-I in adult patients, and there are few randomised studies comparing it with alternative supraglottic airway devices [1,4]. Further, we could not find any study on the effect of fibreoptic-guided intubation through Ambu Aura-I on intracranial pressure measured invasively or indirectly using changes in optic nerve sheath diameter (ONSD) measurement as a surrogate for invasive ICP measurement during anaesthesia.

We, therefore, hypothesised that airway management and tracheal intubation through Ambu Aura-I may be associated with lesser changes in ONSD compared to conventional tracheal intubation and designed a study to ultrasonographically measure the changes in optic nerve sheath diameter following tracheal intubation using Macintosh laryngoscope or fibreoptic-guided intubation through Ambu Aura-I in patients receiving endotracheal anaesthesia.

## Materials and methods

This randomised controlled hospital-based clinical study was conducted on 60 patients divided into two groups, undergoing elective surgery under general anaesthesia requiring tracheal intubation. Institutional ethical committee and institutional research review board approval were obtained (reference number: MGMCH/IEC/JPR/2019/32). Thereafter, the study was registered with the Clinical Trials Registry of India (www.ctri.nic.in) (reference/registration identity: CTRI/2020/09/027798). Recruitment of the patients for the study was started after the registration with Clinical Trial Registry of India (CTRI) was obtained.

Inclusion criteria were of an adult patient of American Society of Anesthesiologists (ASA) Grade I/II willing to participate of aged 18-55 years with weight 50 to 70 kg, undergoing elective surgery requiring tracheal intubation. Exclusion criteria were of patients unwilling with systemic diseases causing compromised organ function (ASA Grades III, IV, and V) and having predicted difficult airway, mouth opening less than 2.5 cm or abnormal airway indices or intraoral growth. The patient with obesity body mass index (BMI) >35 kg/m^2^, hiatus hernia, gastroparesis, pregnancy, or trauma, with known or allergic to any drugs used in the protocol, were also excluded from the study.

Baseline parameters (heart rate, blood pressure, and end-tidal carbon dioxide concentration [EtCO_2_]) before induction of anaesthesia were recorded for further comparison. ONSD was thereafter measured by transorbital sonography with the patient lying in the supine position using a portable Sonosite Turbo-M ultrasonography (Fujifilm Sonosite, Bothell, USA) machine. ONSD measurement was done `in the transverse and coronal plane in both eyes. After applying Tegaderm (3M, Gurugram, Haryana) over the closed eyes, a thick layer of coupling gel was applied over it over the closed upper eyelid. The ultrasound high-frequency probe was placed gently without exerting pressure, and axial images of the orbit were acquired in the plane of the optic nerve. The structures of the eye were visualised in order to visualise the optic nerve directly opposite to the probe, with the ONSD width perpendicular to the vertical axis of the examined plane. Baseline ONSD (before induction of anaesthesia) was measured 3 mm behind the globe in both eyes. Observation of EtCO_2_ at this time was also recorded.

A standard technique of anaesthesia was followed. When the neuromuscular block was complete, the patients were assigned group allocation for airway management technique to either group 1 or group 2 using a computerised random numbers: group 1 (n=30, tracheal intubation facilitated by direct laryngoscopy with Macintosh laryngoscope) or group 2 (n=30, fibreoptic-guided tracheal intubation through Ambu Aura-I).

After tracheal intubation, further maintenance of anaesthesia was done with nitrous oxide and oxygen (50% each) and intermittent rocuronium and isoflurane. At the end of the surgery, anaesthetics were stopped and reversal of residual neuromuscular block was done as per standard practice.

Ambu Aura-I size was chosen according to the manufacturer’s guidance based on body weight (size 3 for patients weighing 30-50 kg, size 4 for 50-70 kg, and size 5 for more than 70 kg). The Ambu Aura-I was inserted as per the technique suggested by the manufacturers. The airway tube of the prechecked and prepared lubricated Ambu Aura-I was held by working hand with three fingers on airway tube and thumb on the vertical line on connector shell (pen insertion technique). The tip was inserted inside the mouth with circular movement maintaining contact against the palate and posterior pharynx wall. The advancement of Ambu Aura-I has culminated as the resistance was felt and the placement of two horizontal lines on the airway tube coincides with patients’ incisors. Once Ambu Aura-I properly was placed, rest of the procedure of cuff inflation with the appropriate volume of air was done and fibreoptic-guided tracheal intubation was accomplished. Removal of Ambu Aura-I was done over a smaller plain tracheal tube used for railroading as per the manufacturers’ recommendation.

ONSD was repeated in both the eyes in all studied patients at the following time intervals: (1) immediately after induction of anaesthesia, (2) five minutes after tracheal intubation, and (3) 10 minutes after intubation, which was the endpoint of the study.

Surgery was allowed to proceed only thereafter. Observations of HR, systolic blood pressure (SBP), diastolic blood pressure (DBP), mean blood pressure (MBP), oxygen saturation (SpO_2_), and EtCO_2_ at these above-mentioned time interval were recorded along with ONSD measurements, and complications were recorded. Data collected were tabulated, and statistical analysis was done using SPSS 22.00 for windows (SPSS Inc, Chicago, USA).

## Results

In our study, both groups were demographically comparable (Table 1). On comparing the haemodynamic variables, the heart rate and systolic, diastolic and mean blood pressure before induction of anaesthesia were comparable in both the groups (Table 2). Endotracheal intubation in both groups resulted in an increase in heart rate and blood pressure. The trend of changes in heart rate in group 2 was similar to group 1 in acquiring statistical significance. 

**Table 1:** Demographic data ASA: American Society of Anesthesiologists.

Variables	Group 1		Group 2		Chi-square value/t	p-value
Age in years (mean±SD)	31.98	5.86	31.77	5.94	0.170	0.072
Gender						
Female [number (%)]	27	90%	28	93%	0.218	0.640
Male [number (%)]	3	10%	2	7%		
Weight in kg (mean±SD)	66.03	10.78	64.33	10.81	0.370	0.540
Height in cm (mean±SD)	168.23	6.55	166.53	6.54	1.010	0.320
ASA Grade						
I [number (%)]	27	90%	25	83%	0.577	0.447
II [number (%)]	3	10%	5	17%		

**Table 2:** Haemodynamic changes HR: heart rate, SBP: systolic blood pressure, DBP: diastolic blood pressure, MAP: mean arterial pressure. *p<0.05.

Variables	Group 1		Group 2		t-test	p-value
HR in beats/min	Mean	SD	Mean	SD		
Before induction	79.53	6.07	81.53	6.07	1.630	0.210
Immediately after intubation	90.20	6.42	84.07	5.59	24.520	<0.01*
Five minutes after intubation	92.07	6.09	85.20	6.42	27.300	<0.01*
10 minutes after intubation	95.67	6.09	86.07	6.09	31.890	<0.01*
SBP in mm Hg						
Before induction	126.10	7.078	128.08	8.04	1.190	0.280
Immediately after intubation	132.09	7.01	137.11	4.81	9.88	0.003*
Five minutes after intubation	135.53	5.34	139.53	4.98	2.69	0.12*
10 minutes after intubation	137.84	4.81	142.727	5.58	2.87	0.08*
DBP in mm Hg						
Before induction	78.87	5.81	80.08	5.14	2.100	0.150
Immediately after intubation	86.07	6.07	84.93	5.84	2.470	0.100
Five minutes after intubation	88.19	6.02	86.17	6.31	1.970	0.230
10 minutes after intubation	87.27	5.38	86.91	5.47	1.740	0.370
MAP in mm Hg						
Before induction	94.70	5.05	96.79	5.62	1.310	0.460
Immediately after intubation	95.367	5.74	97.99	5.80	1.680	0.280
Five minutes after intubation	96.30	5.45	98.09	5.74	1.020	0.410
10 minutes after intubation	97.63	5.33	97.04	5.43	0.390	0.620

Changes in optic nerve sheath diameter have been displayed in Tables 3, 4. In our study, ONSD value before anaesthesia administration was 3.87±0.34 mm in the right eye and 3.86±0.34 mm in the left eye in group 1 (control group), whereas it was 3.62±0.32 mm in the right eye and 3.58±0.33 mm in the left eye in group 2 (intubation guided by fibreoptic laryngoscopy using Ambu Aura-I as a conduit). The mean ONSD before intubation was similar in both groups (p>0.05).

**Table 3:** ONSD changes in right eye ONSD: optic nerve sheath diameter.

Group		ONSD (right)						
		Before induction	Immediately after intubation	Five minutes after intubation	10 minutes after intubation	Before vs immediate	Before vs five minutes	Before vs 10 minutes
Group 1	Mean	3.873	4.27	4.36	4.03	0.48	0.32	0.59
	SD	0.349	0.445	0.49	0.32			
Group 2	Mean	3.62	3.98	4.19	4.06	0.52	0.37	0.31
	SD	0.332	0.492	0.352	0.298			
t-test		0.91	0.78	0.70	0.19			
p-value		0.26	0.45	0.51	0.82			

**Table 4:** ONSD changes in left eye ONSD: optic nerve sheath diameter.

Group		ONSD (left)						
		Before induction	Immediately after intubation	Five minutes after intubation	10 minutes after intubation	Before vs immediate	Before vs five minutes	Before vs 10 minutes
Group 1	Mean	3.867	4.0	4.37	4.02	0.71	0.34	0.63
	SD	0.345	0.309	0.42	0.37			
Group 2	Mean	3.58	3.93	4.16	4.05	0.41	0.29	0.35
	SD	0.330	0.49	0.314	0.298			
t-test		0.89	0.78	0.70	0.19			
p-value		0.25	0.45	0.51	0.82			

The ONSD value was 4.27±0.44 mm in the right eye and 4.0±0.30 in the left eye immediately after intubation in group 1. An increase in ONSD value from a baseline value of the order of >10% (compared to before induction) was recorded immediately after intubation. The ONSD further increased and peaked at five minutes after intubation (4.36±0.49 and 4.37±0.42 mm in right and left eyes, respectively). The ONSD decreased slightly to 4.03±0.32 and 4.02±0.37 mm in right and left eyes, respectively, at 10 minutes after intubation. The mean values though appeared pretty more than the before induction value, the ONSD changes were, however, not statistically different in multiple comparisons within the group 1 (p>0.05).

The trend in ONSD changes in group 2 was also similar to group 1. The before intubation ONSD was 3.62±0.33 and 3.58±0.33 mm in right and left eyes, respectively. An increase of 9% over the baseline value was recorded after fibreoptic-guided intubation through Ambu Aura-I. The ONSD increase peaked at 4.19±0.35 and 4.16±0.31 mm in right and left eyes, respectively. Like in group 1, the ONSD decreased slightly to 4.06±0.29 and 4.05±0.29 mm in right and left eyes, respectively, in group 2 at 10 minutes after intubation. The changes in ONSD when compared to baseline values (before intubation) were statistically not significant (p>0.05). Between-group comparison in ONSD in both the eyes at different time intervals was statistically not significant (p>0.05).

## Discussion

Our observations on haemodynamic effects of tracheal intubation are in agreement with those observed by Kihara et al. in both groups [2]. They suggest that the pressor response to direct laryngoscopy (DL) and endotracheal intubation precipitating a significant increase in heart rate and systemic blood pressure is an established phenomenon and thus a cause of concern for anaesthesiologists all over. The majority of studies treat laryngoscopy and intubation as a single stimulus, which manifestly they are not. The mechanism of haemodynamic response to laryngoscopy and orotracheal intubation is proposed to be by somato-visceral reflexes. Stimulation of proprioceptors at the base of the tongue during laryngoscopy induces impulse-dependent increases of systemic blood pressure, heart rate, and plasma catecholamine concentrations. Subsequent orotracheal intubation recruits additional receptors that elicit augmented haemodynamic and epinephrine responses as well as some vagal inhibition of the heart.

When ICP rises, cerebrospinal fluid (CSF) is pushed towards the tiny rim of subarachnoid space between the sheath and the nerve, causing an expansion of the dural covering. These changes are more marked within the anterior part of the nerve sheath behind the globe. As with any physiological change, the ONSD changes dynamically with changes in ICP [3].

Optic nerve sheath ultrasound is a simple, safe, inexpensive, and bedside diagnostic assay analogous to the measurement of BP and has the potential to exchange invasive ICP monitoring in cases of raised intracranial hypertension. Ophthalmic ultrasound basically uses a frequency between 5 and 10.5 MHz to evaluate the eye and the orbit. At this point (3 mm behind the globe), any changes in the intracranial pressure can be conveniently measured by transocular sonography, which has now become standard of care for noninvasive monitoring of intracranial pressure and has been found to be having fairly good association as far as sensitivity and specificity is concerned. Ultrasonographically measured ONSD is thus considered to be a good surrogate for ICP [4-8].

A Chinese study by Wang et al. studied ONSD changes after laryngoscopy with Macintosh laryngoscope and subsequent tracheal intubation and compared it with intubation after intratracheal surface anaesthesia airway management with LMA alone [9]. They observed an increase in ONSD immediately after and five minutes after intubation with Macintosh laryngoscope. Their observations validate the trend observed in our study. Although the Chinese authors recorded a statistically significant difference, their study is silent on concomitant EtCO_2_ changes observed at measured time intervals, unlike our study. Kucusoman et al. (2020) studied ONSD changes after intubation with three different types of the laryngoscope [10]. The Macintosh laryngoscope and McCoy laryngoscope resulted in an increase in ONSD, whereas the C-Mac videolaryngoscope did not result in an increase in optic nerve sheath diameter.

In fact, there is a paucity of literature regarding the clinical use of intubating LMA or Ambu Aura-I regarding the effect of intubating laryngeal mask airway (ILMA) insertion or intubation through it on changes in ONSD or ICP. An elaborate search in the Medical Subject Headings (MESH) database with keywords intubating LMA, Ambu Aura-I, optic nerve sheath diameter, and intracranial pressure did not yield any result. Ferson shared his experience based on an isolated case report, wherein a patient with posterior fossa mass with raised intracranial pressure required an emergency craniotomy [11]. Ventriculostomy was performed for the initial decrease in intracranial pressure. He thereafter used LMA for airway management to minimise the intracranial pressure responses and found that oxygenation followed by hyperventilation resulted in a decrease in intracranial pressure. He then performed fibreoptic-guided intubation through LMA to minimise the exaggerated effect on intracranial pressure and recorded a marginal rise of only 16-18 mm Hg in ICP following fibreoptic intubation through LMA.

Limitations of the study

Firstly, we have not used the gold standard (invasive intracranial pressure monitoring) for the measurement of ICP. Although the direct technique of measuring ICP such as ventriculostomy is considered a standard technique but is invasive and troublesome to perform routinely in all suspected patients of raised ICP. Ultrasonographic ONSD measurement is a noninvasive method and claims a good correlation with ICP. In this manner, ONSD proves to be a valuable surrogate in the evaluation and diagnosis of raised ICP without any complications associated with the invasive methods. Secondly, we did not study intubation time and therefore are unable to know the influence of the difference in induction-intubation time which has been different in the study groups due to differences in the technique of tracheal intubation.

## Conclusions

On the basis of this study, we conclude that fibreoptic-guided tracheal intubation through Ambu Aura-I is not superior to tracheal intubation using direct laryngoscopy with Macintosh laryngoscope in terms of its effect on intracranial pressure, as measured ultrasonographically by optic nerve sheath diameter.
